#  A Reversed Phase High Performance Liquid Chromatographic Method for Determination of Rapamycin 

**Published:** 2013

**Authors:** Hamideh Sobhani, Alireza Shafaati, Nastaran Nafissi-Varcheh, Reza Aboofazeli

**Affiliations:** a Department of Pharmaceutics, School of Pharmacy, Shahid Beheshti University of Medical Sciences, Tehran, Iran.; b Department of Pharmaceutical Chemistry, School of Pharmacy, Shahid Beheshti University of Medical Sciences, Tehran, Iran.; c Department of Pharmaceutical Biotechnology, School of Pharmacy, Shahid Beheshti University of Medical Sciences, Tehran, Iran.

**Keywords:** Rapamycin, Sirolimus, RP- HPLC, UV detection

## Abstract

Easily degradating and various isomeric forms of rapamycin (Sirolimus) face the determination of this compound to many challenges. In this study, we developed and validated the isocratic reversed phase high performance liquid chromatographic (RP-HPLC) method for rapamycin. Separation was performed on a C_8 _column (MZ, 15 × 4.6 mm, 5 μm particle size) using methanol:water (80:20 v/v) as the mobile phase with the flow rate of 1 mL/min. The column temperature was set at 57°C and the detection was carried out at the wavelength of 277 nm. The method was linear over a concentration range of 0.025-2 μg/mL. The coefficient of variation of intra- and inter-day, assessed at three concentration levels of 0.075, 0.3 and 0.900 μg/mL, was less than 2%. Limit of quantification (LOQ) was found 25 ng/mL. The method with high percent recovery and short retention time of rapamycin, was found to be simple, rapid and reproducible.

## Introduction

**Figure 1:**
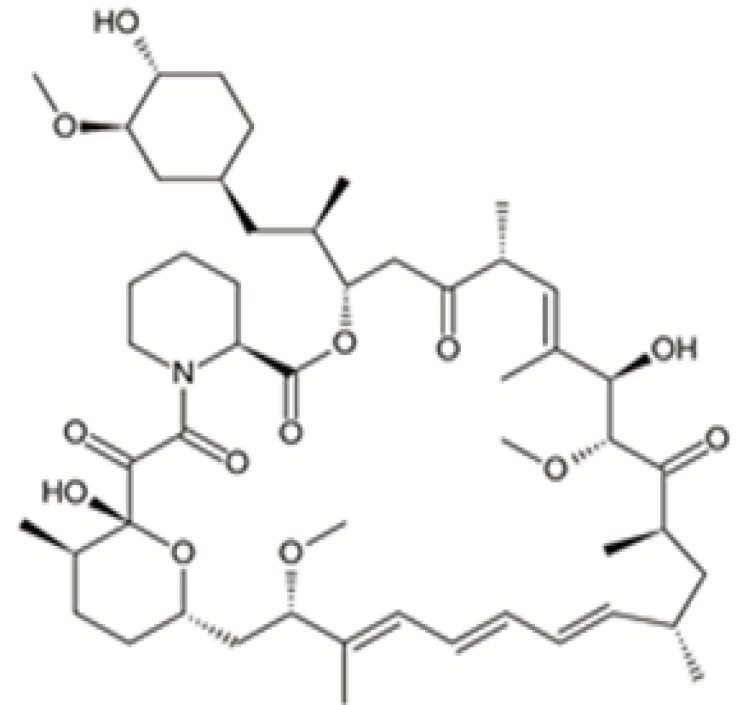
Chemical structure of rapamycin

Rapamycin (Sirolimus, RAP) (Figure 1), a carboxylic lactone-lactam macrolide antibiotic, was first extracted from *Streptomyces hygroscopicus *from soil samples of Easter Island with efficacious anti-candida, immunosuppressive and anti-proliferative effects (1, 2). RAP was approved as an immunosuppressive by the US Food and Drug Administration with the brand name of Rapamune to restraint of renal transplant rejection. 

Ring cleavage is a typical reaction that RAP undergoes under a variety of experimental conditions. Ring-opened compounds, also known as seco-derivatives, exhibit extremely weak immunosuppressive activity (3). RAP exists as a mixture of two conformational isomers in various solutions which are detectable by NMR. The main component is isomer B (*β*) that contains 3-10% and less than 0.5% of isomer C (*γ*) and A (*α*), respectively (4, 5). 

Common analytical methods for the assay of RAP, especially in drug monitoring, are chromatographic separations with ultraviolet detection (4, 6, 7), mass spectrometric detection (8-10) and immunoassay techniques (11, 12). Although these methods are accepted and validated, they usually suffer from a long analysis time or involve equipment (HPLC-MS) not commonly available. On the other hand, feasible, and robust analytical methods are demanded for the assay of RAP in pharmaceutical dosage forms. No report was found in the literature dealing with the determination of RAP in our specifically developed nanoemulsion-based formulations. Thus, in order to fulfill the growing needs on such an analytical method for RAP quality control, we developed and validated a reliable HPLC technique with the potential use for the analysis of the drug when solubilized in the oil phase of o/w nanoemulsion formulations.

## Experimental

*Materials*

HPLC grade methanol, Tween 20 and Triacetin were purchased from Merck Chemical Company (Darmstadt, Germany). Water was prepared by a Millipore Milli-Q Plus Water Purification System. RAP (Batch SIRLI0012) was provided by Zahravi Pharmaceutical Company (Zahravi, Iran). Transcutol was gifted from Gattefosse Company (Gattefosse, France). All other reagents used were of analytical grade.

*Chromatographic system and conditions*

Chromatographic analysis was performed on Knauer HPLC system (Germany), consisting of a pump (Smartline 1000), UV detector (Smartline 2500) and software (Chromgate V3.1.7). A reversed phase C_8 _(MZ, 15 × 4.6 mm, 5 μm particle size, MZ Analysentechnik GmbH, Germany) was used for the separation. A proper guard column (C_8_, 5 μm particle size) was also applied. Mobile phase was a mixture of methanol:water (80:20 v/v) which was freshly prepared and degassed each day. Column temperature was set at 57°C (Knauer, Germany). The injection of the samples was performed on a Reodyne injector equipped with a 20 μL loop. UV detector was set at 277 nm.

*Standard preparation*

A standard stock solution of RAP with the concentration of 10 μg/mL was prepared by dissolving RAP in methanol. The obtained solution was stored at - 20°C. All other solutions (including those used for the construction of the calibration curve and the preparation of quality control samples) were prepared by the dilution of the stock solution with appropriate amount of the methanol.

*Sample preparation*

RAP-loaded nanoemulsion (NE) containing Tween 20 as surfactant, triacetin as oil, 2-propanol and transcutol as co-surfactants with the drug content of 1 mg/mL was prepared by mixing the appropriate amounts of each component. A blank formulation was also prepared to evaluate the possible interferences of the NE base. NE mixtures were diluted 1000 times by adding proper portions of mobile phase to the mixtures, before analysis.

*Degradation product*

In the course of our study, we prepared NE formulation containing RAP, mixed them with NaCl 0.9% w/v solution containing 0.05% w/v Tween 80, and kept them at 37°C for a week. The mixture were then diluted as described in sample preparation and subjected to the HPLC method.

*Method validation*

Method validation was performed according to the accepted guidelines (13-15). Briefly, precision, intra- and inter-day variations, linearity over a specified concentration range and accuracy (measured as percent recovery) were assessed. Linearity was evaluated over a concentration of range of 0.025-2.000 μg/mL at seven different concentration levels. Precision of the method was assessed by the repeated analysis (10 injections) of a solution containing 1 μg/mL of RAP. Inter and intra-day variations and percentage of recovery were determined by six successive injections of three concentration levels (0.075, 0.3 and 0.900 μg/mL) of the drug. Inter-day variation was defined by the repeated analyses performed on three different days and described as the relative standard deviations (RSDs) for each concentration level.

## Results and Discussion

RAP, an immunosuppressive and anti-proliferative drug, is chemically a hydrophobic molecule with very low water solubility (2.6 μg/mL) and no ionizable functional groups over a wide pH range (4). RAP forms two isomeric *β *and *γ *forms in different solvents like acetone, chloroform, dichloromethane and ethyl alcohol (5). Therefore, it is necessary to ensure that analytical method is capable of separating isomeric mixtures. Chromatographic techniques, especially HPLC, were considered as the first choice for RAP analysis. However, as mentioned earlier, HPLC methods usually suffer from long analysis time or involve equipment (*i.e.*, HPLC-MS) not commonly available.

The aim of the main project was to use nanoemulsion as an efficient vehicle for the delivery of RAP. As the first step, we specifically developed our laboratory nanoemulsion formulations loaded with RAP, using appropriate surfactant, oil and cosurfactants. Therefore, the present study was planned to set up an HPLC method potentially capable of analyzing the solubilized drug in the nanoemulsion vehicle. Literature review also showed no reports for the determination of RAP in our specifically developed nanoemulsion formulations.

Initial attempts were made on C_18 _and C_8_ columns to obtain an efficient separation of the analytes, practical retention times and good peak shapes. Separation performed on a C_18_ column showed longer analysis time (more than 10 min) and consumed larger amounts of the organic modifier. The same results have been reported in previously published works (4, 6, 7). Results obtained from the preliminary study in this research revealed that the application of a C_8_ column with less hydrophobic characteristic was advantageous over a C_18_ column, such that the need for methanol as the organic modifier, as well as analysis time were both reduced. Thus, separation process was performed on an MZ-C_8_ column which resulted in reasonable retention times and successful separation of the analytes. The pH of the aqueous portion of the mobile phase was not considered as an important factor since the drug has no ionizable functional groups within the pH range of 1-10 (4).

**Figure 2:**
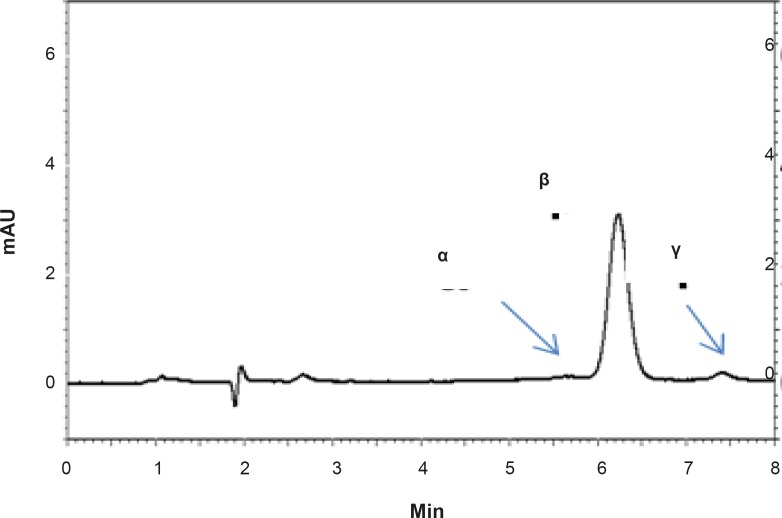
HPLC chromatograms showing the separation of RAP isomers (*α, β *and *γ*) in a standard solution of RAP in methanol.

Methanol was preferred over acetonitrile as an organic phase, since by using methanol, better peak shapes and shorter retention times were obtained. Under optimum chromatographic conditions, tailing factors were found to be 1.12. Figure 2 shows a typical HPLC chromatogram obtained after the injection of a standard solution of RAP.

**Figure 3:**
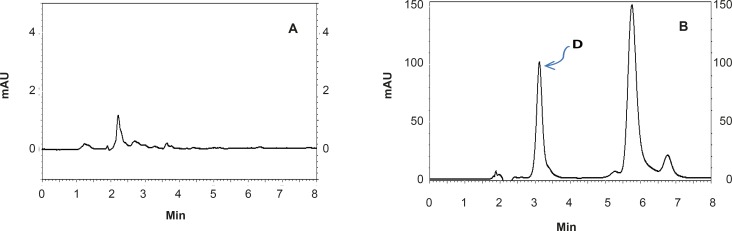
HPLC chromatograms showing: (A) a blank formulation of nanoemulsion diluted with the mobile phase and (B) a RAP-loaded nanoemulsion after one week incubation at 37°C (D shows the degradation product of RAP).

**Figure 4:**
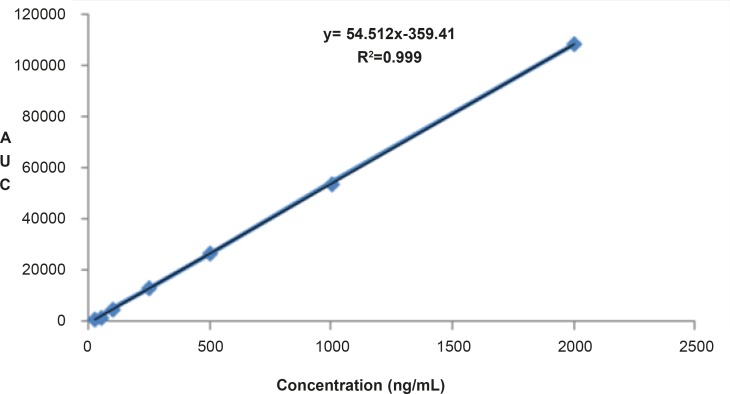
Linear relationship obtained between the analyte peak area and RAP concentrations over a range of 0.025-2.000 μg/mL.

The proposed method was fully validated according to international guidelines (13-15). Blank nanoemulsion was prepared and diluted with methanol (1000 times) and analyzed by the proposed method (Figure 3a). No interfering peak was observed, suggesting the specificity of the method and its suitability for the routine quality control analyses. Adequate resolution between the peaks relevant to RAP beta-isomer (Figure 2) and RAP degradation product (D in Figure 3b) was also observed. Linear relationship was obtained between the analyte peak area and the corresponding concentrations over a wide range of 0.025-2.000 μg/mL (R^2 ^= 0.999, Y = 54.512x - 359.41) (Figure 4). The value of the relative standard deviation (RSD) for the precision assessment was calculated to be 0.93% (n = 10). The results expressed as RSDs, obtained from intra- and inter- day assessment (presented as percent of recovery) are given in Table 1 and the results of accuracy testing is given in Table 2. Figure 3 indicates that the proposed method was successful for the analysis of RAP in NE formulation with no interferences from the components used in the vehicle.

**Table 1:** Results of intra- and inter-day variation assessment expressed as RSD% (n = 3).

**Parameters**	**results**		
	concentration (μg/mL)		
	0.075	0.3	0.9
Intra-day	0.18	1.2	1.4
Inter-day	0.27-1.23	0.33-1.45	0.35-1.3

**Table 2:** Accuracy assessment expressed as percent of recovery (n = 3).

**Parameter**	**results**		
	concentration (μg/mL)		
	0.075	0.3	0.9
Recovery	83.8	77.6	80.3

## Conclusion

In this work, a fast and reliable HPLC method for the identification and quantification of RAP was developed and validated. 
